# First-Trimester Uterine Artery Doppler Indices and Pregnancy Outcomes in Naturally Conceived and Frozen–Thawed Embryo Transfer Cycles

**DOI:** 10.3390/diagnostics15172223

**Published:** 2025-09-02

**Authors:** Elif Ganime Aygün, Edis Kahraman

**Affiliations:** Acibadem Mehmet Ali Aydinlar University, Atakent Hospital, Istanbul 34303, Türkiye; ediskahramani@gmail.com

**Keywords:** assisted reproductive technology (ART), dydrogesterone, micronized progesterone, Doppler, uterine artery pulsality index, frozen embryo transfer

## Abstract

**Background/Objectives**: The role of luteal phase support (LPS) in frozen–thawed embryo transfer (FET) cycles has garnered increasing interest, particularly regarding its influence on uterine perfusion and pregnancy outcomes. This study aimed to investigate the effect of different oral LPS regimens on first-trimester uterine artery Doppler indices and their association with early pregnancy outcomes in naturally conceived and FET pregnancies. **Methods**: This retrospective cohort study included 289 singleton pregnancies comprising spontaneous conceptions, FET cycles supported with oral micronised progesterone, and FET cycles supported with oral dydrogesterone. The uterine artery pulsatility index (PI) was measured via Doppler ultrasound during the first trimester. Group comparisons were performed using non-parametric tests. Multivariable regression analyses were used to assess independent predictors of PI and associations with gestational diabetes and low birth weight. **Results**: Uterine artery PI values differed significantly among the groups (*p* < 0.001). The lowest PI was observed in the dydrogesterone group, followed by the naturally conceived and micronised progesterone groups. A higher maternal body mass index (BMI) was independently associated with lower PI (*p* = 0.009), while maternal age showed no significant effect. No significant associations were found between PI or maternal characteristics and adverse outcomes such as gestational diabetes or low birth weight. **Conclusions**: Dydrogesterone was associated with more favorable uterine artery Doppler indices in early pregnancy, suggesting improved uteroplacental adaptation in FET cycles. These findings support further prospective research to determine the clinical impact of LPS regimens on placental development and perinatal health.

## 1. Introduction

Assisted reproductive technology (ART) has undergone substantial advancements in recent decades, with frozen–thawed embryo transfer (FET) now comprising an increasingly prominent share of treatment cycles globally [1]. Improvements in vitrification techniques have resulted in high embryo survival rates and clinical outcomes following FET that are comparable to—or in some cases superior to—those of fresh embryo transfers, contributing to a widespread shift in clinical practice [2,3].

In FET cycles, especially those utilizing artificial endometrial preparation protocols that lack a functional corpus luteum, luteal phase support (LPS) with exogenous progesterone is essential for achieving endometrial receptivity and maintaining early pregnancy [4,5]. LPS can be administered through various routes, including vaginal, oral, intramuscular, rectal, and subcutaneous formulations [6,7]. Among oral formulations, micronised progesterone and dydrogesterone acetate are widely prescribed due to their ease of administration and favorable safety profiles [8,9]. Randomized controlled trials and meta-analyses have shown that oral dydrogesterone achieves pregnancy rates comparable to those of standard vaginal progesterone, supporting its use as an alternative in FET cycles [7,10].

Beyond hormonal supplementation, successful pregnancy also depends on appropriate uteroplacental vascular adaptation [11]. The uterine artery pulsatility index (PI), measured during the first trimester via Doppler ultrasound, serves as a non-invasive indicator of uterine vascular resistance [12]. Elevated PI has been linked to impaired placentation and an increased risk of adverse outcomes such as preeclampsia, fetal growth restriction, and other pregnancy complications [13,14,15].

Maternal characteristics may also modulate uteroplacental perfusion and pregnancy outcomes. Elevated body mass index (BMI) has consistently been associated with heightened risks of gestational diabetes, placental dysfunction, and abnormal fetal growth trajectories, including both macrosomia and, in some cases, growth restriction [16,17]. Advanced maternal age, likewise, is a well-established risk factor for hypertensive disorders, fetal growth restriction, and adverse perinatal outcomes, independent of the mode of conception [18,19].

In light of these factors, the present study aimed to compare first-trimester uterine artery PI and pregnancy outcomes among women who conceived spontaneously and those who conceived through FET cycles supported with either oral micronised progesterone or dydrogesterone. Additionally, we examined the relationships between uterine artery PI, maternal characteristics, and early pregnancy outcomes across these groups.

## 2. Materials and Methods

### 2.1. Study Design and Ethical Approval

This retrospective cohort study was conducted at the Assisted Reproductive Techniques Center of Acıbadem Atakent Hospital and included women who conceived between January 2021 and December 2023. The study protocol received approval from the Acıbadem Atakent Hospital Ethics Committee on 11 January 2024 (Approval No: 2023-21/749) and adhered to the principles outlined in the Declaration of Helsinki. Written informed consent was obtained from all participants for the anonymized use of their clinical data for research purposes.

### 2.2. Participant Selection

Eligible participants were women aged 34 to 40 years who achieved pregnancy either spontaneously or through FET. Based on the mode of conception and type of LPS, participants were stratified into three groups: (1) spontaneously conceived pregnancies (natural conception group); (2) FET cycles supported with oral micronized progesterone (Progestan 200 mg, Koçak Farma, Istanbul, Türkiye); and (3) FET cycles supported with oral dydrogesterone acetate (Duphaston 20 mg, Abbott Laboratories, Istanbul, Türkiye). Importantly, all FET pregnancies in this study were conceived following artificial endometrial preparation using hormone-replacement therapy (HRT) protocols. Natural-cycle FETs were intentionally excluded to ensure a uniform hormonal environment across the treatment groups. This approach allowed for a more accurate evaluation of the effect of oral progesterone formulations on uterine artery Doppler indices during early pregnancy.

The indications for infertility treatment among women who underwent FET included female factor infertility, male factor infertility, unexplained infertility (such as tubal occlusion, diminished ovarian reserve, abnormal semen parameters, or failure to conceive despite one year of unprotected intercourse), and elective embryo cryopreservation for personal or medical reasons.

Participants were excluded if they had received alternative LPS regimens, had multiparity (defined as ≥2 prior live births), or presented with multiple gestations. Further exclusion criteria included a documented history of preterm birth, endometriosis, polycystic ovary syndrome (PCOS), ovarian insufficiency, or chronic medical conditions such as pregestational diabetes mellitus, chronic hypertension, or malignancy.

Women with PCOS were excluded due to the known endocrine and metabolic abnormalities associated with the condition, which may independently affect uterine blood flow and early placental development. Similarly, ovarian insufficiency was defined based on documented clinical diagnoses in patient records and supported by elevated serum follicle-stimulating hormone (FSH) levels (>25 IU/L), low anti-Müllerian hormone (AMH < 0.5 ng/mL), or antral follicle count (AFC) <5 follicles. Although a universally accepted diagnostic threshold for ovarian insufficiency is lacking, the ESHRE Guideline Development Group (GDG) recommends using these parameters to identify diminished ovarian reserve. Exclusion of these conditions aimed to reduce confounding factors related to abnormal endometrial receptivity or perfusion.

Women diagnosed with gestational diabetes mellitus (GDM) during pregnancy, based on a 75 g oral glucose tolerance test (OGTT), were not excluded, as GDM was considered one of the study outcomes. Participants with incomplete clinical data or who delivered outside the study institution were excluded to minimize data inconsistency and ensure uniform follow-up.

### 2.3. Data Collection and Clinical Assessments

Maternal demographic and clinical information was extracted from electronic medical records. First-trimester evaluations included maternal age, BMI, thyroid-stimulating hormone (TSH) levels, hemoglobin (Hb), hematocrit, platelet count, and uterine artery Doppler indices. Uterine artery PI was measured via transabdominal Doppler ultrasound using a Voluson E8 Expert device (General Electric, Chicago, IL, USA) by a single experienced obstetrician (E.G.A.) during routine first-trimester screening. Cervical length measurements were available for all participants; however, they were excluded from the final analysis as they were not directly relevant to the primary study endpoints involving uterine artery Doppler indices and early placental development in low-risk pregnancies. TSH levels were quantified via electrochemiluminescence immunoassay using the COBAS 8000 e801 analyzer (Roche Diagnostics GmbH, Mannheim, Germany), and complete blood counts were obtained using the Sysmex XN-1000 hematology analyzer (Sysmex Corporation, Kobe, Japan). GDM was diagnosed in accordance with national guidelines using the 75 g OGTT. Perinatal outcomes, recorded only for participants who delivered at the study hospital, included gestational age at delivery, neonatal birth weight and length, placental weight, and total maternal gestational weight gain.

### 2.4. Statistical Analysis

All statistical analyses were conducted using GraphPad Prism software (version 10.0; GraphPad Software, San Diego, CA, USA). Normality of continuous variables was assessed using the Shapiro–Wilk test, which indicated non-normal distributions across all parameters; accordingly, non-parametric methods were applied throughout.

Continuous variables were reported as medians with interquartile ranges (IQRs), while categorical variables were expressed as frequencies and percentages. Comparisons of continuous variables across the three study groups (e.g., maternal age, BMI, PI values) were performed using the Kruskal–Wallis test, followed by Dunn–Bonferroni post hoc analysis when significant differences were observed.

Spearman’s rank correlation coefficients were used to evaluate associations between uterine artery PI and continuous maternal or perinatal variables, including age, BMI, hemoglobin, hematocrit, platelet count, birth weight, and placental weight. Categorical outcomes such as GDM incidence were compared between groups using the chi-square test or Fisher’s exact test, depending on cell counts.

Multivariable linear regression analysis was conducted to identify independent predictors of uterine artery PI, incorporating maternal age, BMI, and type of LPS as covariates. Additionally, logistic regression models were employed to explore associations between PI, maternal characteristics, and adverse outcomes such as GDM or low birth weight. Results are presented as beta coefficients or odds ratios (ORs) with corresponding 95% confidence intervals (CIs). A two-tailed *p*-value < 0.05 was considered statistically significant.

## 3. Results

A total of 289 pregnant women were included in the study, comprising 72 (24.9%) spontaneous conceptions, 92 (31.8%) pregnancies following FET supported with oral dydrogesterone acetate, and 125 (43.3%) FET pregnancies supported with oral micronized progesterone. Regarding gestational age at delivery, 7.6% delivered at 36 weeks, 11.8% at 37 weeks, 42.2% at 38 weeks, 34.9% at 39 weeks, and 3.5% at 40 weeks. The incidence of GDM was 16.6%, while 83.4% of participants remained GDM-negative.

Baseline characteristics and pregnancy-related parameters, stratified by treatment group, are presented in Table 1. The median maternal age was 36.0 years (IQR 34.0–40.0) in the dydrogesterone group, 36.0 years (IQR 33.0–40.0) in the micronized progesterone group, and 37.0 years (IQR 34.0–40.0) in the spontaneous conception group. There was no statistically significant difference in maternal age across groups (*p* = 0.148). The median BMI was lowest in the dydrogesterone group at 22.84 kg/m^2^ (IQR 17.37–29.04), followed by the spontaneous conception group at 24.26 kg/m^2^ (IQR 18.60–26.90), and highest in the micronized progesterone group at 23.31 kg/m^2^ (IQR 16.49–32.56). This difference was statistically significant (*p* = 0.014). Post hoc analysis using the Dunn–Bonferroni method revealed significantly higher BMI in both the spontaneous conception group (*p* = 0.014) and the micronized progesterone group (*p* = 0.004) compared to the dydrogesterone group.

**Figure 1:**
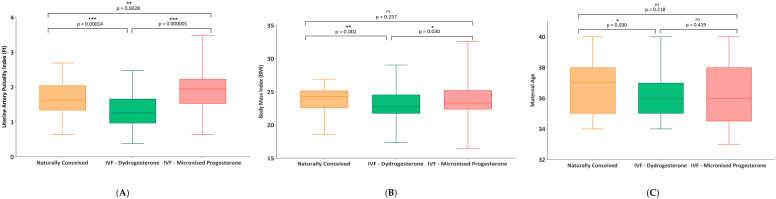
Comparison of key maternal parameters across treatment groups. (**A**) Uterine artery PI, (**B**) BMI, and (**C**) maternal age distributions by treatment group. Asterisks indicate significant pairwise differences (* *p* < 0.05, ** *p* < 0.01, *** *p* < 0.001).

Significant differences were also observed in first-trimester uterine artery PI across groups (*p* < 0.001). The lowest median PI was observed in the dydrogesterone group (1.26; IQR 0.38–2.48), followed by the spontaneous conception group (1.62; IQR 0.64–2.69), and the highest in the micronized progesterone group (1.95; IQR 0.64–3.48). Post hoc comparisons confirmed that PI values were significantly lower in the dydrogesterone group compared to both the spontaneous conception (*p* = 0.0001) and micronized progesterone groups (*p* < 0.000001), while the spontaneous conception group also showed significantly lower PI compared to the micronized progesterone group (*p* = 0.0028) (Figure 1). These findings indicate that the type of luteal phase support significantly influences early uterine vascular adaptation, with dydrogesterone acetate associated with more favorable Doppler profiles.

**Figure 2:**
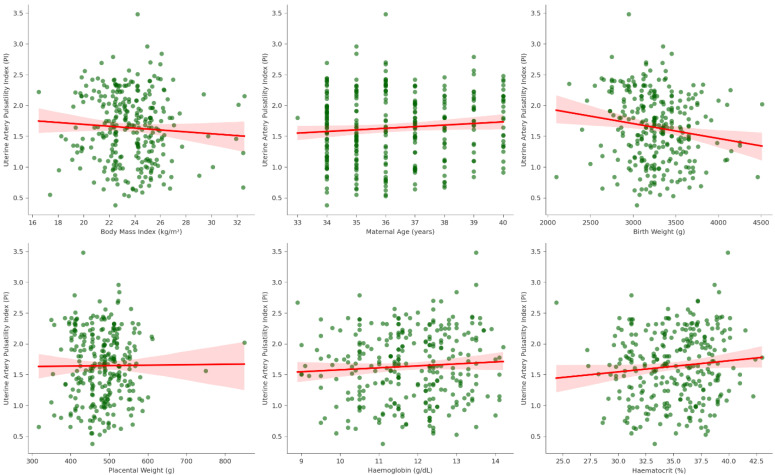
Correlation analyses between uterine artery PI and maternal/perinatal variables.

Spearman correlation analyses identified a weak but statistically significant inverse association between BMI and uterine artery PI (ρ = –0.18, *p* = 0.002), suggesting that higher BMI was associated with lower uterine artery resistance. No significant correlations were found between PI and maternal age (ρ = –0.07, *p* = 0.218), birth weight (ρ = –0.09, *p* = 0.141), placental weight (ρ = –0.01, *p* = 0.869), hemoglobin (ρ = 0.05, *p* = 0.413), hematocrit (ρ = 0.05, *p* = 0.415), or platelet count (ρ = –0.04, *p* = 0.541) (Figure 2).

To further investigate independent predictors of uterine artery PI, a multivariable linear regression model was constructed including maternal age, BMI, and LPS type. BMI remained a significant independent negative predictor of PI (β = –0.035, *p* = 0.009). Both micronized progesterone (β = 0.553, *p* < 0.001) and spontaneous conception (β = 0.307, *p* < 0.001) were associated with significantly higher PI compared to dydrogesterone. Maternal age did not independently predict PI (*p* = 0.131) (Table 2). Finally, logistic regression analyses assessed whether PI, maternal age, or BMI predicted GDM or low birth weight (<2500 g). None of the variables were significantly associated with GDM (PI: OR 1.40, *p* = 0.254; age: OR 1.08, *p* = 0.327; BMI: OR 1.09, *p* = 0.201) or with low birth weight (PI: OR 1.20, *p* = 0.846; age: OR 1.32, *p* = 0.275; BMI: OR 1.11, *p* = 0.624).

## 4. Discussion

This study examined the effects of different LPS strategies on uterine artery resistance and early pregnancy outcomes in women who conceived either spontaneously or through FET. Our findings indicate that the type of progesterone used in FET cycles significantly influences uterine artery PI. In particular, oral dydrogesterone was associated with the lowest PI values compared to both micronized progesterone and spontaneous conception, suggesting a more favorable hemodynamic profile and potentially enhanced uterine perfusion in early gestation.

These results align with previous research indicating that both the mode of conception and the type of LPS can impact uterine artery Doppler parameters [20,21]. A lower uterine artery PI is generally considered a surrogate marker of improved uterine blood flow and successful spiral artery remodeling—key processes necessary for optimal placental development [22,23,24,25,26,27,28]. However, in the context of assisted reproduction, this relationship becomes more complex. For example, a recent systematic review by Zaat et al. [29] reported that although artificial endometrial preparation in FET cycles may reduce PI, it does not necessarily translate into reduced risks of hypertensive disorders or abnormal placentation, suggesting that reduced vascular resistance alone does not ensure favorable outcomes [21,30,31,32,33].

Dydrogesterone has been extensively studied for its role in promoting implantation and enhancing endometrial receptivity, with randomized controlled trials demonstrating outcomes comparable to vaginal progesterone in IVF settings [34,35]. However, its influence on uteroplacental vascular dynamics has been less thoroughly characterized. Emerging data suggest that dydrogesterone may have a favorable effect on uterine blood flow parameters [36,37], and our results reinforce this hypothesis. Compared to micronized progesterone and spontaneous conception, dydrogesterone appears to support a more favorable vascular environment in early pregnancy. This is consistent with literature proposing that different progesterone formulations exert distinct physiological effects on vascular tone and endometrial function [38,39]. One plausible explanation for these differences lies in the molecular actions of dydrogesterone. The compound has been shown to modulate immune signaling and cytokine expression within the endometrium, shifting the maternal immune response toward an anti-inflammatory, pregnancy-supportive profile—an environment conducive to vascular remodeling [40,41,42].

Although elevated PI has been associated with adverse outcomes such as preeclampsia and fetal growth restriction [13,14,15], our study did not find significant associations between PI and gestational diabetes or low birth weight. This may reflect the low-risk nature of the study population and the modest sample size. Similar findings have been reported in prior studies suggesting that in low-risk cohorts, uterine artery PI is not strongly predictive of gestational metabolic complications or fetal growth anomalies [43,44]. Nonetheless, the early PI differences observed across LPS groups may hold clinical relevance in higher-risk populations where vascular maladaptation is more prevalent [45,46].

An inverse association was also observed between maternal BMI and uterine artery PI, indicating that higher BMI was linked to lower vascular resistance. This finding is consistent with previous studies reporting that maternal adiposity can influence uteroplacental hemodynamics [47,48]. However, while lower PI might suggest improved perfusion, obesity is independently associated with abnormal placentation, preeclampsia, and adverse fetal growth trajectories [49,50,51]. Thus, interpretation of Doppler indices must account for maternal metabolic status and comorbidities.

Maternal age was not a significant predictor of PI in our analysis, consistent with studies suggesting that the age-related risks to pregnancy may arise through mechanisms other than impaired uterine perfusion [28,52,53].

Cervical length is another parameter commonly assessed during pregnancy, primarily for predicting preterm birth in the second trimester. Although this measurement was available in our dataset, it was not included in the final analysis, as current evidence suggests that cervical length in the first trimester has limited clinical relevance to uteroplacental perfusion or vascular adaptation, particularly in low-risk or ART populations [54].

All FET cycles in our study were performed using HRT protocols with artificial endometrial preparation, and natural-cycle FETs were not included. This design choice was made to ensure a standardized hormonal milieu and eliminate variability introduced by spontaneous ovulation and endogenous progesterone production. The type of endometrial preparation is known to influence endometrial receptivity and vascular adaptation, and previous studies have shown that outcomes may differ between natural-cycle and HRT-FET protocols. By restricting our cohort to HRT-FET cycles, we aimed to isolate the effect of oral progesterone formulations on uterine artery Doppler parameters and early pregnancy outcomes.

We deliberately excluded women with PCOS and ovarian insufficiency to minimize confounding effects stemming from hormonal or metabolic disturbances that could independently alter uterine blood flow. Although these conditions are common in infertility, our aim was to isolate the effect of luteal phase support in a more hormonally balanced population. Future studies with stratified designs should explore whether these findings hold in PCOS or low-responder subgroups.

In summary, our findings suggest that oral dydrogesterone may confer a vascular advantage in early pregnancy among women undergoing FET, potentially supporting more favorable uteroplacental adaptation. These results highlight the importance of carefully selecting LPS strategies in ART, not only for implantation support but also for their potential downstream effects on uterine vascular physiology. Nevertheless, this study is subject to limitations, including its retrospective design, single-center scope, and moderate sample size. Furthermore, only oral progesterone regimens were assessed; vaginal and combined approaches warrant future investigation. Despite these constraints, our results contribute to a growing body of evidence suggesting that the type of progesterone used in LPS protocols may influence early gestational hemodynamics. Larger prospective studies are essential to confirm these findings and assess their implications for long-term perinatal outcomes.

## 5. Conclusions

This study highlights that the type of LPS administered during FET cycles significantly impacts uterine artery resistance in early pregnancy. Oral dydrogesterone was associated with lower uterine artery PI values compared to both oral micronised progesterone and spontaneous conception, suggesting a more favorable hemodynamic environment and potentially enhanced uteroplacental perfusion. Although uterine artery PI did not correlate with adverse outcomes such as gestational diabetes or low birth weight in this low-risk cohort, the observed early vascular differences may have greater clinical relevance in higher-risk populations. Furthermore, maternal BMI emerged as an independent determinant of uterine artery resistance, emphasizing the importance of considering maternal metabolic factors when interpreting Doppler findings. Collectively, these findings support the potential utility of tailoring progesterone regimens in assisted reproduction to improve early vascular adaptation. Further large-scale, prospective studies are needed to determine whether such hemodynamic improvements contribute to enhanced placental development and perinatal outcomes.

## Figures and Tables

**Table 1:** Demographic characteristics, pregnancy measurements, and birth outcomes by treatment group.

*DEMOGRAPHIC CHARACTERISTICS*								
	***N***	**%**		***N***	**%**		***N***	**%**
**Agent**			**Birth week (week)**			**GDM**		
Naturally Conceived group	72	24.90%	36	22	7.60%	Present	48	16.60%
IVF-Dydrogesterone group	92	31.80%	37	34	11.80%	Absent	241	83.40%
IVF-Micronised Progesterone group	125	43.30%	38	122	42.20%			
			39	101	34.90%			
			40	10	3.50%			
*PREGNANCY MEASUREMENTS AND BIRTH OUTCOMES BY TREATMENT GROUP*								
	**Naturally Conceived group**		**IVF-Dydrogesterone group**		**IVF-Micronised Progesterone group**			
	**Mean (SD)**	**Median (Min–Max)**	**Mean (SD)**	**Median (Min–Max)**	**Mean (SD)**	**Median (Min–Max)**		
**Age (year)**	36.68 (1.93)	37.0 (34–40)	36.03 (1.75)	36.0 (34–40)	36.34 (2.08)	36.0 (33–40)		
**BMI (kg/m^2^)**	23.92 (1.91)	24.26 (18.6–26.9)	22.97 (2.2)	22.84 (17.37–29.04)	23.93 (2.71)	23.31 (16.49–32.56)		
**TSH level (mIU/L)**	1.56 (0.84)	1.35 (0.4–3.75)	1.82 (1.02)	1.72 (0.45–6.2)	1.79 (0.82)	1.75 (0.45–4.52)		
**Cervical length (mm)**	36.81 (3.87)	36.2 (30.2–51.4)	36.02 (2.81)	35.7 (30.4–44.1)	36.52 (3.14)	36.2 (29.8–46.3)		
**Haematocrit level (%)**	35.45 (3.36)	36.1 (24.4–43.0)	34.88 (3.12)	34.85 (28.6–42.2)	34.92 (3.16)	35.35 (27.2–42.4)		
**Ultrasound day (days)**	38.15 (1.06)	38.0 (36–40)	38.25 (0.79)	38.0 (36–40)	38.07 (0.98)	38.0 (36–40)		
**Haemoglobin level (g/dL)**	11.86 (1.19)	12.05 (8.9–14.1)	11.79 (1.15)	11.7 (9.0–14.1)	11.76 (1.21)	11.75 (9.0–14.2)		
**Platelet count × 10^3^ (cell/mL)**	231.64 (62.65)	221.5 (123.0–435.0)	232.82 (65.24)	225.5 (119.0–433.0)	226.19 (74.12)	211.0 (109.0–458.0)		
**Average PI**	1.64 (0.5)	1.62 (0.64–2.69)	1.33 (0.5)	1.26 (0.38–2.48)	1.86 (0.52)	1.95 (0.64–3.48)		
**Placenta weight (g)**	481.74 (49.91)	488.0 (375.0–578.0)	486.38 (69.36)	483.0 (357.0–850.0)	479.13 (57.63)	487.0 (317.0–612.0)		

**Table 2:** Multivariable linear regression analysis of predictors for uterine artery pulsatility index (PI).

Explanatory Variable	β Coefficients	95% CI	*p* Value	Significance
BMI	−0.035	−0.061 to −0.009	0.0090	**
Maternal Age	−0.018	−0.042 to 0.006	0.1310	NS
Micronised Progesterone (vs. Dydrogesterone)	0.553	0.401 to 0.705	0.0001	***
Naturally Conceived (vs. Dydrogesterone)	0.307	0.195 to 0.419	0.0001	***

** *p* < 0.01, *** *p* < 0.001, NS, not significant.

## Data Availability

The data presented in this study are available upon request from the corresponding author.

## Abbreviations

The following abbreviations are used in this manuscript:

ART	Assisted Reproductive Technology
BMI	Body Mass Index
CI	Confidence Interval
FET	Frozen–Thawed Embryo Transfer
GDM	Gestational Diabetes Mellitus
Hb	Hemoglobin
Hct	Hematocrit
HRT	Hormone-Replacement Therapy
IQR	Interquartile Range
IVF	In Vitro Fertilization
LPS	Luteal Phase Support
OR	Odds Ratio
PI	Pulsatility Index
PCOS	Polycystic Ovary Syndrome
OGTT	Oral Glucose Tolerance Test
TSH	Thyroid-Stimulating Hormone
